# High-quality science requires high-quality open data infrastructure

**DOI:** 10.1038/sdata.2018.27

**Published:** 2018-02-27

**Authors:** Susanna-Assunta Sansone, Patricia Cruse, Mark Thorley

**Affiliations:** 1 University of Oxford, Oxford e-Research Centre, Department of Engineering Sciences, Oxford OX1 1TQ, UK; 2 DataCite, Welfengarten 1B, Hannover 30167, Germany; 3 STFC Rutherford Appleton Laboratory, Scientific Computing, Harwell Campus, Didcot OX11 0QX, UK

**Keywords:** Research management, Databases

## Abstract

Resources for data management, discovery and (re)use are numerous and diverse, and more specifically we need data resources that enable the FAIR principles^1^ of Findability, Accessibility, Interoperability and Reusability of data.

This rolling collection presents a series of open data resources and tools, both new and long-standing, and provides an outlet for the developers and maintainers of these resources to emphasize the approach they take to ensure the data they host and serve are increasingly FAIR. Our interest is not in the purely technical aspect of the work. We are keen to emphasize the positive impact these resources have on the research communities they serve. Last but not least, this collection celebrates developers, data curators, informaticians, and other professionals behind such infrastructure; crediting the often invisible work of designing, developing, curating, maintaining and evolving these resources. The iterative R&D phase of these data resources is not just a technical challenge, but also a social and economic one, which also depends on the size, type, breadth and depth of the data in scope, and the requirement of the community that it serves.

As infrastructures that support the research cycle (from data collection, processing, analysis, presentation, publication, preservation and reuse), the ultimate goal of these resources is to contribute to the process that turns data into knowledge and knowledge into solutions for society’s most pressing challenges. A successful data resource is therefore one that works with and for the relevant research community it serves, by meeting current research needs, as well as providing new opportunities for future research. The first four articles of this collection encompass repositories, tools and services for managing data, presented as digital research assets in their own right, with their own associate research and development life cycle, from design, to development and maintenance.

Bhattacharya *et al*.^2^ describe the enhancement of ImmPort, an established community resource that is one of the largest open and curated repositories of subject-level human immunology data. They highlight their community efforts to formulate and implement the guidelines and standards that enable data sharing and maximize the potential of data reuse and meta-analysis. Akram and colleagues^3^ present the enhancements of NeuroMorpho.Org, the largest public inventory of cellular reconstructions in neuroscience. The authors show how a mature resource must also continue to implement new functionality to increase the efficiency of data curation or the machine-readability of the data. Kugler and Fitch^4^ provide updates on the Integrated Public Use Microdata Series (IPUMS) resource, which for the last 35 years has represented international census and survey data on health, employment, and other topics, in an interoperable and accessible manner. Although the data landscape and technical infrastructure has changed since its first launch, the article is an example of the forward looking attitude towards data annotation and interoperability that drives a successful resource. Lastly, Torre *et al*.^5^ describe a new developed system that helps users to rapidly find datasets, tools, and pre-generated analyses. They also pilot the evaluation of these digital objects against the FAIR principles, storing and displaying the results as an insignia near each dataset, tool, or canned analysis.

Our hope is that the exemplars presented in this rolling collection will contribute to the growing ecosystem of resources working to increase the FAIRness of data. How measurable these improvements are, however, is still an open debate. Hopefully soon we will also be able to assess the level of FAIRness of a data resource as an increased adherence to measurable set of indicators, as proposed by the FAIRmetrics.org group^6^. The resulting FAIRness assessments for the data resources will be stored and displayed in the FAIRsharing registry^7^, a cross-disciplinary resource interlinking repositories, standards and data policies; FAIRsharing, will also provide source information on metadata, identifier schemas and other standards, which are core element to the FAIR principles.

A tremendous variety of data resources exists across the disciplines, at local, national and intercross-national level, driven by large organizations, or taking place within projects, institutes or programmes. We welcome further submissions to this collection from producers and maintainers of such resources.

## Additional information

**How to cite this article:** Sansone, S.-A. *et al.* High-quality science requires high-quality open data infrastructure. *Sci. Data* 5:180027 doi: 10.1038/sdata.2017.27 (2018).

**Publisher’s note:** Springer Nature remains neutral with regard to jurisdictional claims in published maps and institutional affiliations.
